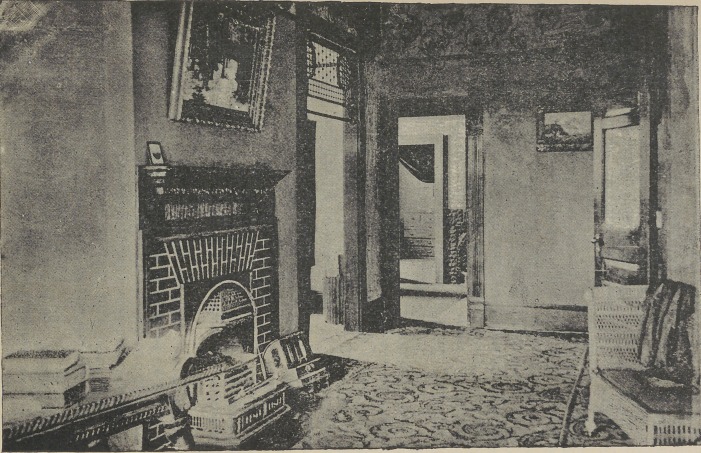# Office Plans

**Published:** 1891-09

**Authors:** 


### Office Plans.

To the readers of the REGISTER the plans of the office of Dr. Geo. H. Wilson, of Painesville, O., is presented for their consideration.

As will be seen by a comparison with the plans of the office published in the June number the arrangement is somewhat different. It is with pleasure too that a photo-engraving of the interior of Dr. Wilson's office is published, for that will lead to a better idea of the plan.

The office is situated at the corner of two of the main thoroughfares and occupies almost the entire second story of a large brick edifice. The building faces the north-western point of the compass, but the operating chairs have a light from the north-east. Entrance to Dr. Wilson's office is made by an easy stairway and as the door is opened the operating chair may be noticed directly opposite the door. From this chair every one entering may be recognized. In the engraving of the interior the reader will notice a heavy curtain at the entrance of the operating room which may be used to obscure the patient seated in the chair. As one proceeds toward the main part of the reception room a heavy oak mantel-piece with fire-place add beauty to the room as well as comfort.

It is difficult to get a photograph showing all the office, so the camera was placed in such a position that the working plan of the office may be better comprehended. Appropriate and tasteful furniture, pictures and bric a brac adorn the reception room evidencing cultured taste. An elegant carpet covers the floor.

SINK

CLOSET

LABORATORY

HALL

2ND OPERATING ROOM

OPERATING ROOM

DRESSING ROOM

RECEPTION ROOM

In the engraving will be noticed that the door is open and a large ground plate glass bearing the name "Wilson" in the door indicates the locality of the office.

Referring to the plan again it will be seen that the operating room and dressing room are separated by a partition of oak wood. The fire of the reception room is arranged to give heat into the dressing room, and in very cold weather a gas-stove may be attached to a pipe that has been arranged for that purpose in case of necessity. The dressing room is large enough also for a couch and office desk.

By a heavy walled partition the extrating and impression rooms are separated from the main rooms. From this there is a wide hall at the end of which is the water closet. Beyond the extracting room is the laboratory, large and commodious. When the doors and curtains are opened all callers may be seen entering from each room by the operator. A door opens from the dressing room into the reception room. A neat fire-place may be noticed in the engraving in the extracting room. By devices similar to the one in the reception room the fire of the extracting room heats the laboratory. The woodwork, doors, windows, etc., are of oak, natural finish. The windows extend a foot from the floor to within a few inches from the ceiling and are wide and have adjustable curtains to regulate the light. Dr. Wilson uses a suspension engine with power from a water-motor. The same motor is used to run the lathe in the laboratory. A sixteen-foot steel shaft extends under the floor from the motor to the operating chair and is regulated with the foot pressing an iron pin that comes through the floor at the chair.

It is almost impossible to do Dr. Wilson justice by this short description but we hope that this will aid to some extent the study of the plan and engraving of his admirable office.

The editors of the REGISTER are indebted to Dr. Wilson for the permission to publish these plans. W.

---

BY reference to advertisement it will be seen that Dr. C. S. Stockton has become the manager of the electro deposit plate Co. For many cases this is the best dental plate made and we are glad that a practical man like Dr. Stockton has taken hold of it. Give it a trial and satisfy yourself as to its merits.

## Figures and Tables

**Figure f1:**
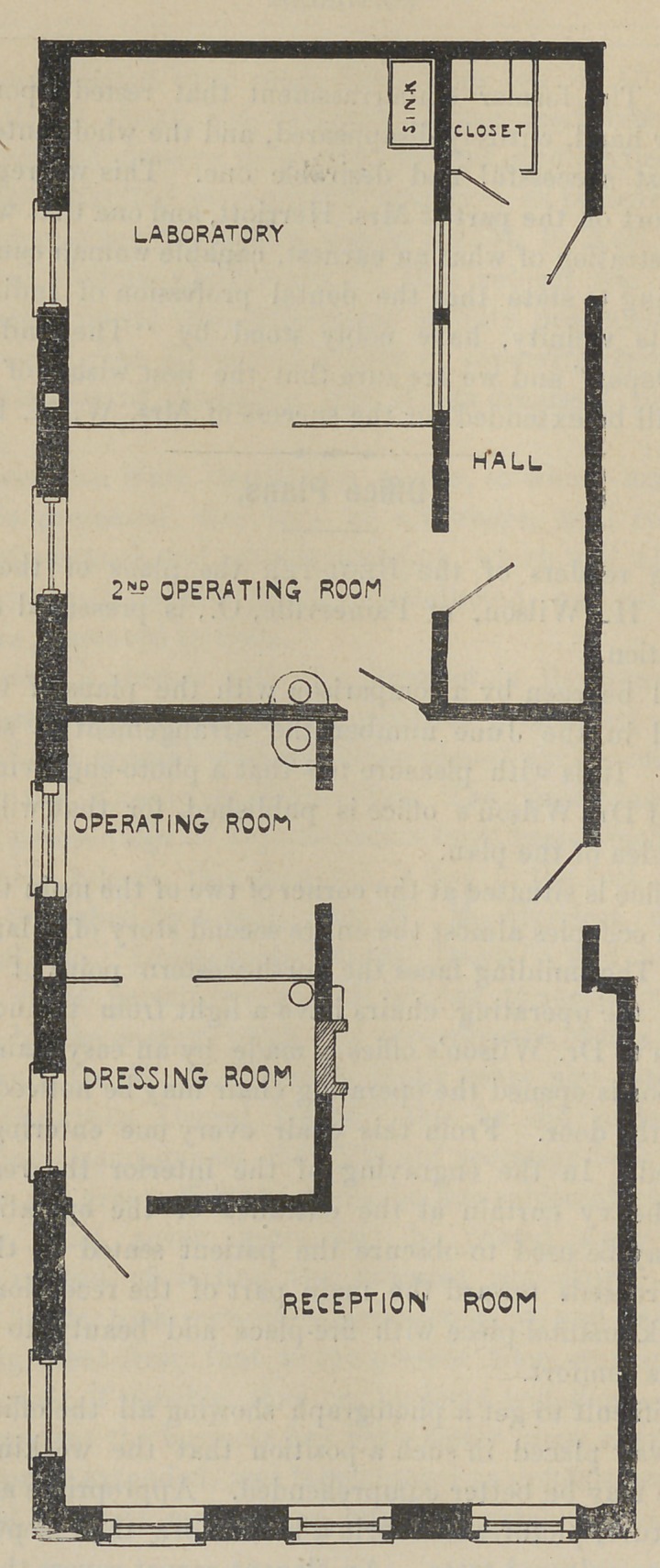


**Figure f2:**